# Evaluation using a four-dimensional imaging tool before and after pulmonary valve replacement in a patient with tetralogy of Fallot: a case report

**DOI:** 10.1186/s13256-018-1964-9

**Published:** 2019-02-05

**Authors:** Masao Takigami, Keiichi Itatani, Naohiko Nakanishi, Kosuke Nakaji, Yo Kajiyama, Satoaki Matoba, Hitoshi Yaku, Masaaki Yamagishi

**Affiliations:** 1 Department of Cardiovascular Medicine, Kyoto Prefectural University of Medicine, 465 Kajii-cho, Kawaramachi-Hirokoji, Kamigyo-ward, Kyoto, 602-8566 Japan; 2 Department of Cardiovascular Surgery, Kyoto Prefectural University of Medicine, Kyoto, Japan; 3 Department of Radiology, Kyoto Prefectural University of Medicine, Kyoto, Japan; 4 Department of Pediatrics, Kyoto Prefectural University of Medicine, Kyoto, Japan; 5 Department of Pediatric Cardiovascular Surgery, Kyoto Prefectural University of Medicine, Kyoto, Japan

**Keywords:** Tetralogy of Fallot, 4D flow MRI, Flow energy loss, Pulmonary regurgitation, Right ventricular deterioration

## Abstract

**Background:**

Pulmonary regurgitation is a common complication after tetralogy of Fallot repair, resulting in right ventricular dysfunction, arrhythmia, and sudden death. However, the indications and optimal timing for pulmonary valve replacement are not fully known. We describe a case in which a four-dimensional imaging tool was useful in the decision to re-operate, thus resulting in decreased energy loss and improved right ventricular function after the re-operation for tetralogy of Fallot.

**Case presentation:**

A 54-year-old Japanese woman visited our hospital due to palpitations and wide QRS tachycardia with persistent tiredness for several months. She underwent repair of tetralogy of Fallot when she was 2-years old. An electrocardiogram showed prolonged QRS duration (199 msec) with a complete right bundle branch block and an echocardiograph demonstrated that her right ventricle was highly enlarged and had poor contraction, and severe pulmonary valve regurgitation with one leaflet flail. Four-dimensional flow magnetic resonance imaging demonstrated that regurgitant volumes and regurgitant fractions of pulmonary regurgitation were calculated as 63.12 ml and 54.0%, respectively. Right ventricular end-diastolic/end-systolic volume index was 169.54/99.76 mL/m^2^, and the cardiac index was 1.78 L/minute per m^2^. Flow energy loss was 2.93 mW, which is estimated to be three times higher than normal controls. An electrophysiological study showed an intact anterior internodal pathway and a slow pathway just through the outside of the right atriotomy line scar, which is supposed to cause a re-entry circuit. We decided to perform a pulmonary valve replacement and a right maze procedure. A 27 mm bioprosthetic valve was implanted in the native pulmonary annulus with a supra-annular position. Concomitantly, the right maze procedure was performed. A four-dimensional flow magnetic resonance imaging done 3 months later showed that right ventricular end-diastolic/end-systolic volume index had significantly reduced to 85.24/55.41 mL/m^2^ and the cardiac index had increased from 1.78 to 2.58 L/minute per m^2^. Energy loss had greatly improved from 2.93 to 1.48 mW.

**Conclusions:**

A four-dimensional imaging tool was useful in the decision to re-operate, thus resulting in decreased energy loss and improved right ventricular function after the re-operation for tetralogy of Fallot.

**Electronic supplementary material:**

The online version of this article (https://doi.org/10.1186/s13256-018-1964-9) contains supplementary material, which is available to authorized users.

## Introduction

Survival after tetralogy of Fallot (TOF) repair has been improving, but long-term mortality is still unsatisfactory, especially 25 years or more after the repair [1]. Pulmonary regurgitation (PR) is a common complication after TOF repair, resulting in right ventricular (RV) dysfunction, arrhythmia, and sudden death [2, 3]. Therefore pulmonary valve replacement (PVR) should be considered before RV deterioration. However, the indications and optimal timing for PVR are not fully known. Some clinical evidence has led physicians to believe that an overly enlarged RV will not be fully reversible even after a PVR. A parameter that would indicate increased ventricular workload before RV deterioration is necessary to clarify the indication for a PVR.

We describe a case in which a four-dimensional imaging tool was useful in the decision to re-operate, thus resulting in decreased energy loss and improved RV function after the re-operation for TOF.

## Case presentation

A 54-year-old Japanese woman visited our hospital due to palpitations and wide QRS tachycardia with persistent tiredness for several months. She underwent repair of TOF when she was 2-years old. After the repair, no follow-up check was performed. During the period, she gave birth to three children. She underwent atrial flutter ablation (cavo-tricuspid isthmus block line) when she was 50-years old and 51-years old.

On admission, she had wide QRS tachycardia of 180 beats/minute, but it spontaneously converted to sinus rhythm. This paroxysmal wide QRS tachyarrhythmia of a few minutes’ duration was easily observed several times.

**Fig. 1:**
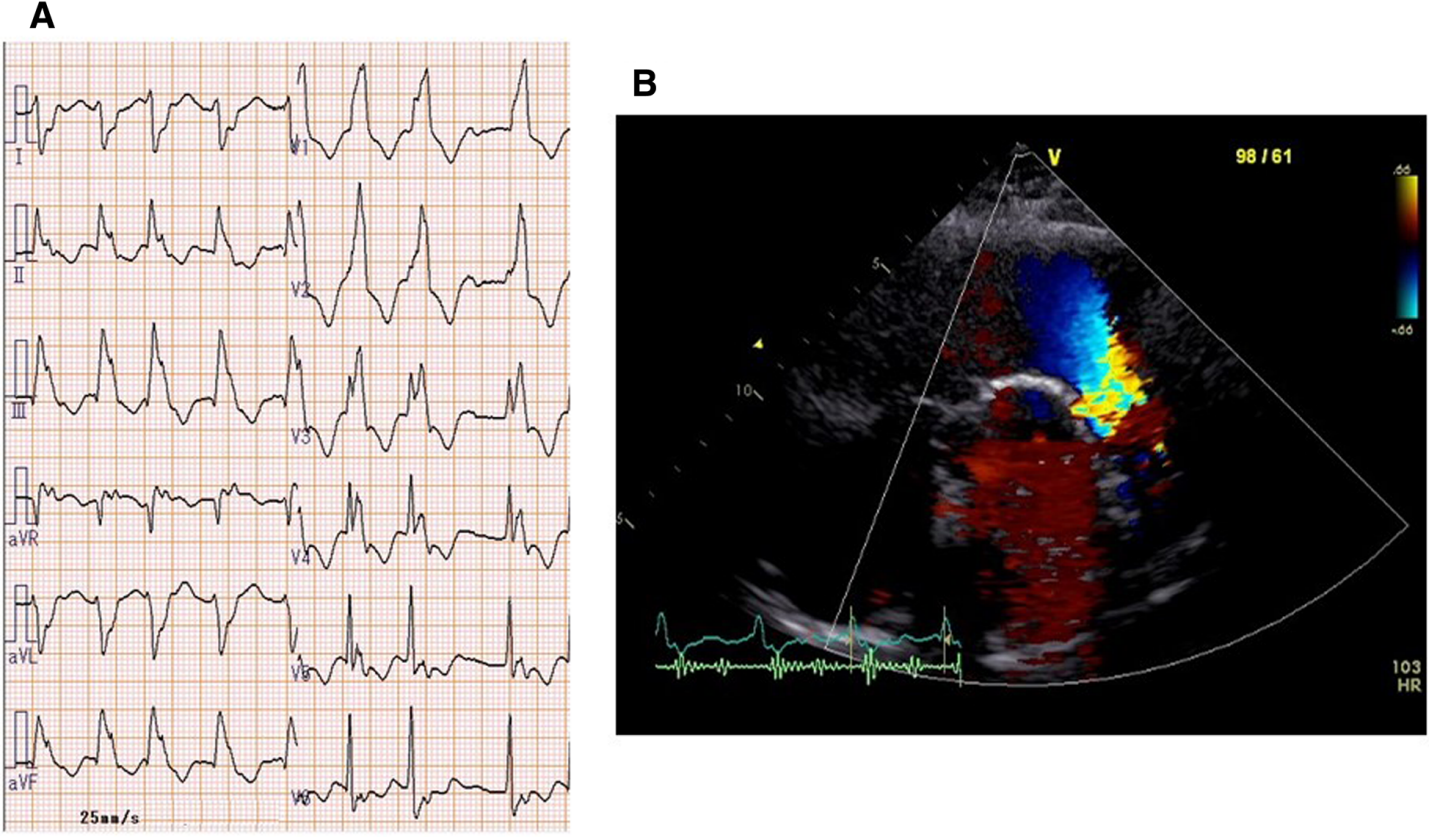
**a** Electrocardiogram. Electrocardiogram showed wide QRS tachycardia (199 msec) with 180 beats/minute. **b** Echocardiograph. Echocardiograph revealed severe pulmonary regurgitation

**Fig. 2:**
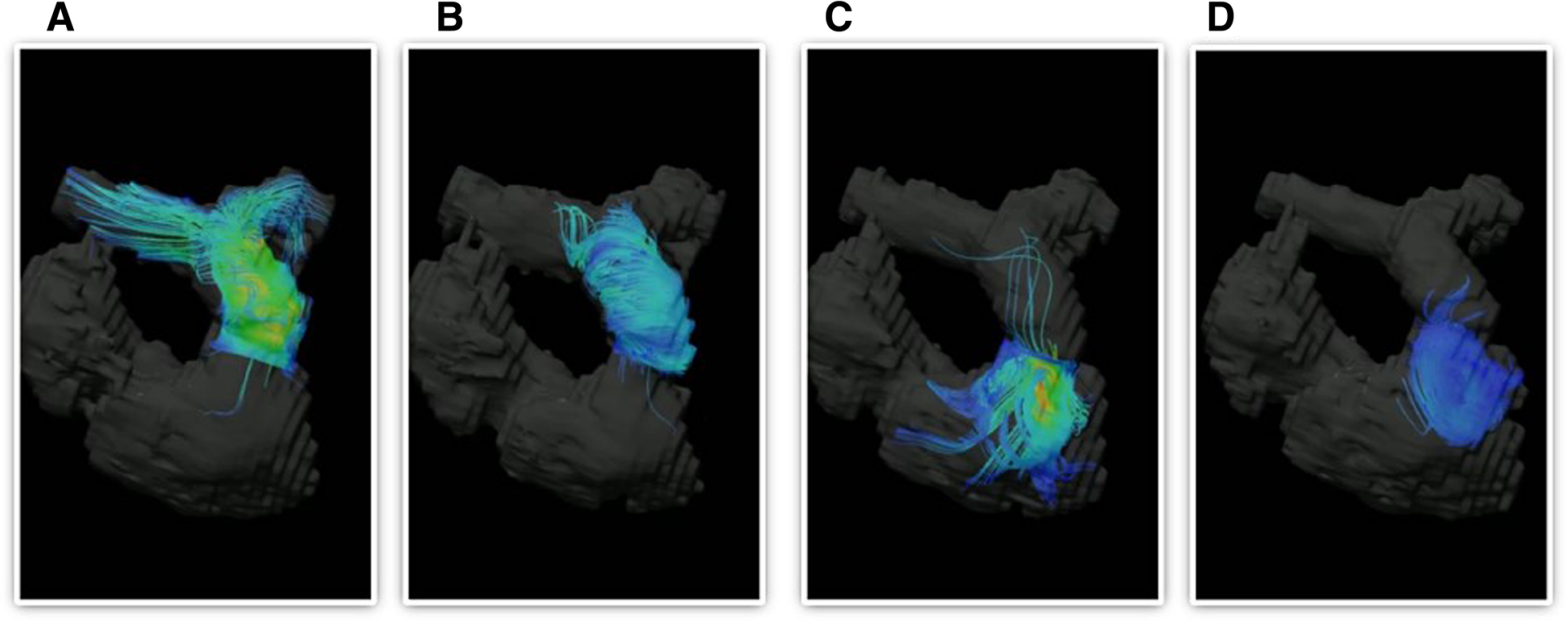
Pulmonary regurgitation of three-dimensional pathline from four-dimensional flow magnetic resonance imaging. **a** early systolic phase, **b** late systolic phase, **c** early diastolic phase, and **d** late diastolic phase

**Fig. 3:**
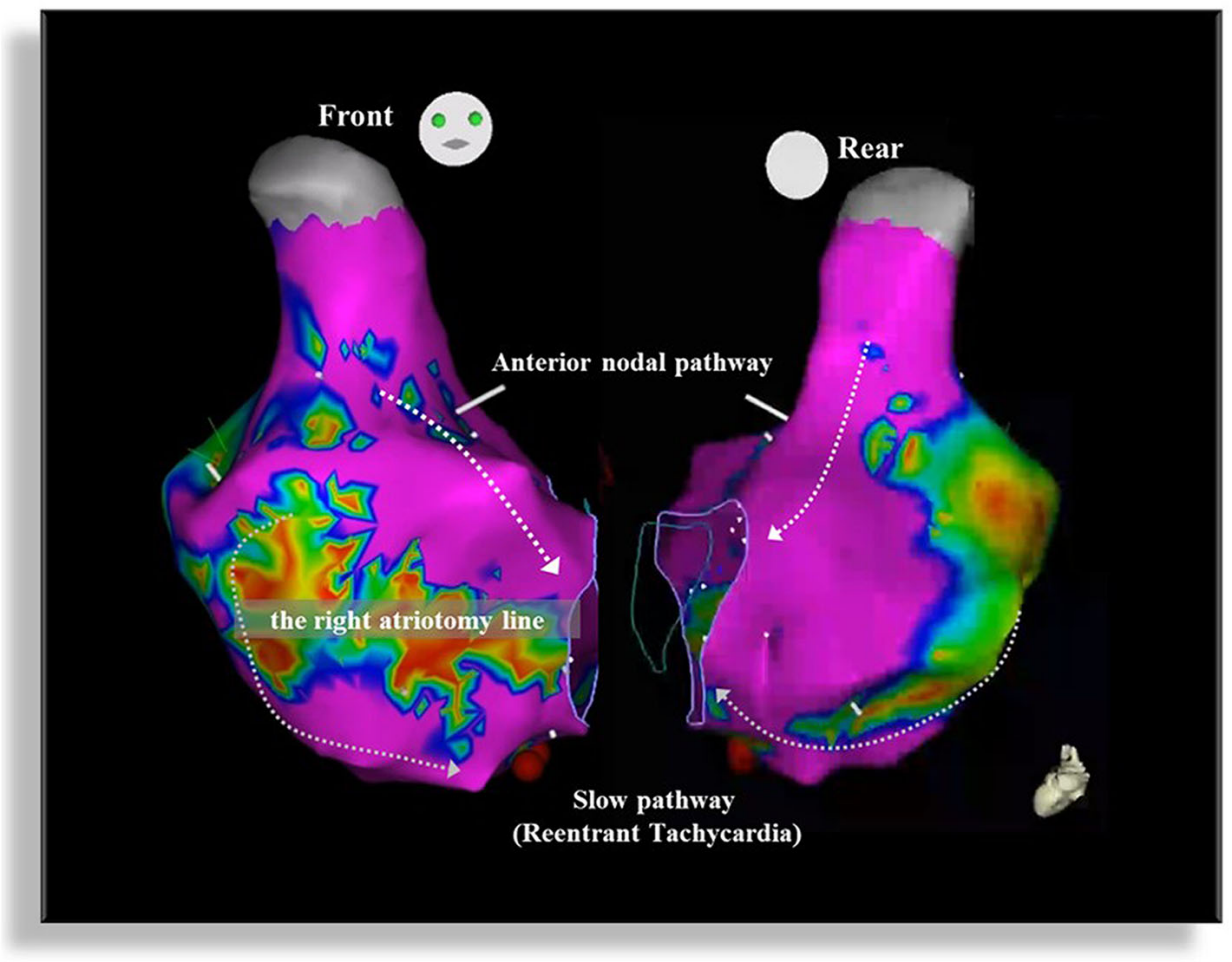
Electrophysiological study. Electrophysiological study showed intact anterior intermodal pathway and slow pathway just through the outside of the right atriotomy line scar, which is supposed to cause re-entry circuit


An electrocardiogram showed prolonged QRS duration (199 msec) with a complete right bundle branch block and an echocardiograph demonstrated that her right ventricle was highly enlarged and had poor contraction, and severe pulmonary valve regurgitation with one leaflet flail (Fig. 1). Four-dimensional flow MRI demonstrated that regurgitant volumes (RVols) and regurgitant fractions (RFs) of PR (Fig. 2 and Additional file 1: Movie S1) were calculated as 63.12 ml and 54.0%, respectively. RV end-diastolic/end-systolic volume index (RVEDVI)/(ESVI) was 169.54/99.76 mL/m^2^, and the cardiac index (CI) was 1.78 L/minute per m^2^. Flow energy loss (FEL) calculated from four-dimensional flow MRI was 2.93 mW, which is estimated to be three times higher than normal controls (Additional file 2: Movie S2). An electrophysiological study showed an intact anterior internodal pathway and a slow pathway just through the outside of the right atriotomy line scar, which is supposed to cause a re-entry circuit (Fig. 3).

Additional file 1:**Movie S1.** Pulmonary regurgitation of 3D pathline from 4D flow MRI imaging. (MP4 8102 kb)



Additional file 2:**Movie S2.** Flow Energy Loss before PVR. (MP4 1096 kb)


We decided to perform a PVR and the right maze procedure because the energy loss of the right side of her heart system was high and, additionally, arrhythmic therapy was needed, even if the extremely dilated RV size did not get restored. Cardiopulmonary bypass (CPB) was instituted with the ascending aorta and bicaval cannulation. Anterior and posterior pulmonary valve leaflets were prolapsed and the left leaflet was highly degenerated. The pulmonary annulus was intact with sufficient size; thus, a bioprosthetic valve was implanted in the native pulmonary annulus with a supra-annular position. After all the leaflets were resected, a Mosaic Ultra® 27 mm (Medtronic, Minneapolis, MN, USA) was placed on the supra-annular position. Concomitantly, the right maze procedure was performed. The right atrium was plicated with the removal of the scar, adding the incision to the lateral side of the atrium, and cryoablation on the posterior and anterior walls of the atrium from the incision line to the tricuspid isthmus and to the inferior vena cava.

**Fig. 4:**
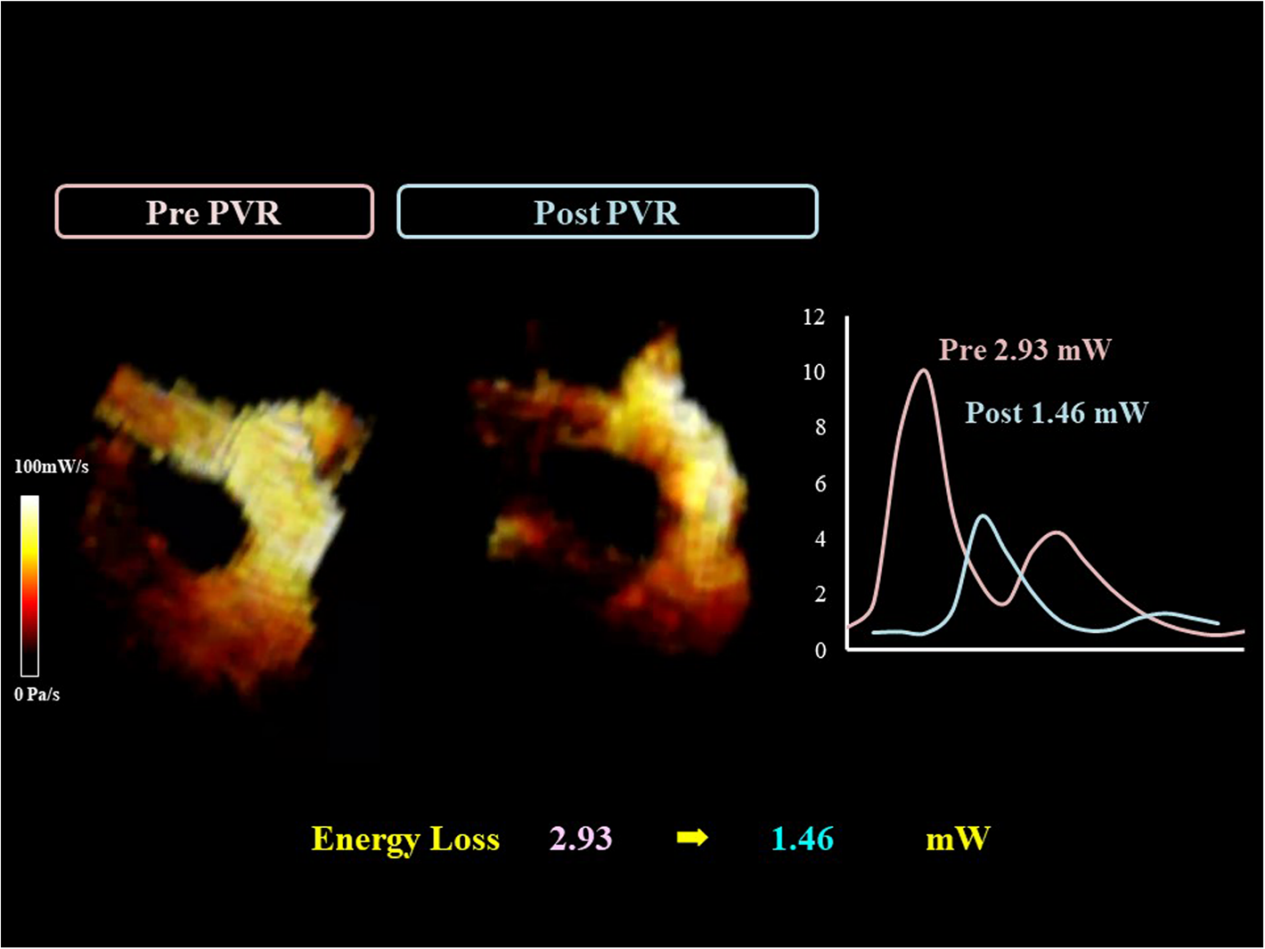
Flow energy loss. Flow energy loss significantly improved from 2.93 to 1.48 mW after pulmonary valve replacement. *PVR* pulmonary valve replacement

Her postoperative course was uneventful and she discharged on day 21. A four-dimensional flow MRI done 3 months later showed that RVEDVI/ESVI had significantly reduced to 85.24/55.41 mL/m^2^ and her CI had increased from 1.78 to 2.58 L/minute per m^2^. Energy loss had greatly improved from 2.93 to 1.48 mW (Fig. 4).

## Discussion

Current surgical correction of TOF has been performed safely, and survival after TOF has been prolonged. However, adult survivors with repaired TOF have late complications such as PR, pulmonary stenosis, tricuspid regurgitation, and RV outflow tract stenosis [1–3]. After TOF repair, patients always have some degree of PR. Longstanding PR makes the RV volume larger, and it will develop into RV dysfunction and a fatal arrhythmia [4]. To avoid further RV deterioration, it is necessary to consider subsequent surgical intervention. Clinical guidelines recommend PVR should be undertaken after RVEDV reaches 170 mL/m^2^ or RVESV reaches 70 mL/m^2^. However, some reports show that RV volumes cannot return to the normal range after PVR if RV volume is too dilated [5]. Thus, we need a useful parameter to be able to predict RV deterioration before RV dysfunction or RV enlargement is developed.

FEL is a useful parameter of cardiac workload and can evaluate and predict ventricular deterioration in myocardial, valvular, and congenital heart disease [6–13]. It has been reported that a FEL calculation based on the total pressure drop on aortic stenosis is predictive of a cardiac event and mortality [14], and it has also been shown that cardiac resynchronization therapy and effective medication therapy for dilated cardiomyopathy reduced energy loss [12, 13, 15]. In the field of congenital heart disease, Shibata *et al.* reported that there were significantly positive correlations between the FEL through the pulmonary valve after TOF repair and the serial change in QRS duration [3].

We calculated the FEL based on viscous dissipation of turbulence using visualized blood flow by four-dimensional flow MRI, which was proven to be equivalent to the FEL measured by total pressure drop [16]. In this case, we considered that RV volume might be irreversible even if we performed re-operation because the RV was too enlarged and the duration of QRS complex was prolonged. However, after the operation, FEL declined greatly and RV size decreased remarkably. This means that the RV workload has been stabilized which contributed to RV reverse remodeling, suggesting that FEL might be a useful tool to consider the timing of re-operation. However, the relationship between the reduction of FEL and reverse remodeling in patients with TOF repair should be further studied.

MRI imaging has excellent reproducibility. Furthermore, some studies suggested MRI is more accurate than echocardiography in assessing the severity of mitral regurgitation (MR) [17], and that RVol by cardiovascular magnetic resonance (CMR) was more predictive of outcomes than transthoracic echocardiography (TTE) in patients with aortic regurgitation (AR) [18]. Considering evaluation of the pulmonary valve, it seems to be more difficult to measure pulmonary RVol correctively by echocardiography. We are convinced that four-dimensional flow MRI is a more effective tool to evaluate the right-sided heart system and to decide optimal timing of the operation.

Arrhythmias are also a common late complication of repaired TOF. Atrial arrhythmias may be present in 65% of patients after TOF repair [2]. Atrial tachyarrhythmias result in left ventricular (LV) dysfunction, leading to systolic heart failure [19, 20]. Some reports have shown that catheter ablation for atrial fibrillation improved LV function and the probability of survival from any cause as compared with the control [21, 22]; however, the arrhythmic involvement in RV dysfunction is still unknown. In this case, the ablation for atrial flutter might have been effective to improve the RV dysfunction after PVR because our patient’s main symptom was caused by arrhythmia rather than by RV dysfunction. Furthermore, because patients with adult congenital heart disease have often undergone several previous surgeries, the cause of arrhythmia is often complicated, and intraoperative ablation is sometimes effective with a mapping guide. Mapping using four-dimensional imaging is useful to treat this type of arrhythmia.

## Conclusion

A four-dimensional imaging tool was useful in the decision to re-operate, thus resulting in decreased energy loss and improved RV function after the re-operation for TOF.
